# Oleic Acid Protects *Caenorhabditis* Mothers From Mating-Induced Death and the Cost of Reproduction

**DOI:** 10.3389/fcell.2021.690373

**Published:** 2021-06-11

**Authors:** Leo S. Choi, Cheng Shi, Jasmine Ashraf, Salman Sohrabi, Coleen T. Murphy

**Affiliations:** Department of Molecular Biology, Lewis-Sigler Institute for Integrative Genomics, Princeton University, Princeton, NJ, United States

**Keywords:** *Caenorhabditis elegans*, longevity, reproduction, oleic acid, mating-induced death, metabolism, cost of reproduction

## Abstract

Reproduction comes at a cost, including accelerated death. Previous studies of the interconnections between reproduction, lifespan, and fat metabolism in *C. elegans* were predominantly performed in low-reproduction conditions. To understand how increased reproduction affects lifespan and fat metabolism, we examined mated worms; we find that a Δ9 desaturase, FAT-7, is significantly up-regulated. Dietary supplementation of oleic acid (OA), the immediate downstream product of FAT-7 activity, restores fat storage and completely rescues mating-induced death, while other fatty acids cannot. OA-mediated lifespan restoration is also observed in *C. elegans* mutants suffering increased death from short-term mating, and in mated *C. remanei* females, indicating a conserved role of oleic acid in post-mating lifespan regulation. Our results suggest that increased reproduction can be uncoupled from the costs of reproduction from somatic longevity regulation if provided with the limiting lipid, oleic acid.

## Introduction

Reproduction is a costly process that depletes energy reserves that may be needed for somatic maintenance and survival (Williams, 1966; Hansen et al., 2013), thus linking reproduction with fat metabolism and lifespan regulation. In many organisms, increased reproduction leads to decreased lifespan, and vice versa (Drori and Folman, 1976; Hansen et al., 2013; Maures et al., 2014; Shi and Murphy, 2014). For example, castrated Korean eunuchs were reported to have lived 15–20 years longer than non-castrated men of similar socio-economic status (Min et al., 2012), while Chinese emperors known for extremely promiscuous behavior lived ∼35% shorter than their counterparts (Shi et al., 2015). *Caenorhabditis elegans* has been established as an outstanding model to investigate these interconnections. In *C. elegans*, removal of germline stem cells leads to a significant increase in lifespan (Hsin and Kenyon, 1999) as well as fat accumulation in the somatic tissues (O’Rourke et al., 2009). By contrast, mating accelerates the proliferation of germline stem cells, increases progeny production, and causes a dramatic fat loss and lifespan decrease (Shi and Murphy, 2014). The genetic analysis of mechanisms underlying the effects of mating or removal of reproductive system on *C. elegans’* fat metabolism and longevity have implicated a network that includes insulin/IGF-1 (Insulin-like Growth Factor-1) signaling, steroid signaling, lipolysis, autophagy, NHR (Nuclear Hormone Receptor) signaling, and fatty acid desaturation (Lapierre and Hansen, 2012). However, the exact relationship between fat loss and lifespan reduction is largely unknown; while fat is generally regarded as its energy source (Hansen et al., 2013), we do not know whether fat is depleted to enhance reproductive outcome, decreasing somatic maintenance and thus shortening lifespan. It also remains unclear exactly which fatty acids are depleted in mated *C. elegans*, and whether different types of fatty acids contribute to different effects in mating-induced death.

Fatty acids are precursor molecules for all lipid classes, including storage lipids [triacylglycerols (TAGs)], membrane lipids (phospholipids and sphingolipids), and signaling lipids (fatty acyl amides, eicosanoids, and others) (Fahy et al., 2009). Fat metabolism plays a crucial role in regulating the lifespan of germlineless mutants and worms with reduced reproduction. Under nutrient-poor and oxidative stress conditions, omega-3 and omega-6 fatty acids mediate the balance of lipid stores between the soma and germline (Lynn et al., 2015), suggesting a pivotal role of fatty acids in coordinating reproduction and somatic aging.

However, these previous *C. elegans* studies examined fat metabolism in animals with no or very low reproduction: either in germlineless mutants, or under conditions such as nutrient deprivation and oxidative stress where reproduction is very limited, or in self-fertilized hermaphrodites limited by sperm number. It remains unclear how fat metabolism changes when reproduction is *increased*, and whether in this circumstance fatty acids still play a role in coordinating somatic aging and reproduction. To address this question, here we increased worms’ reproduction in the most natural way by mating them with males, which typically increases progeny production by 100–200% (Ward and Carrel, 1979; Hodgkin and Barnes, 1991), and examined lifespan, fat levels, transcriptional changes, and supplementation with fatty acids. Remarkably, oleic acid (OA) treatment specifically restores the fat loss induced by mating, and also rescues the lifespan reduction induced by mating, without affecting reproduction. This lifespan rescue by oleic acid supplementation is also pertinent to short-term mating and is conserved in gonochoristic (male and female) *C. remanei* species. Our results suggest that increased reproduction is not associated with inevitable lifespan reduction, and that metabolism of a specific fatty acid is able to uncouple the costs of reproduction from somatic longevity regulation.

## Results

### Mating Induces a Significant Lifespan Decrease Regardless of the Initial Level of Overall Fat Storage

In *C. elegans*, extended longevity is associated with altered fat metabolism and reduced reproduction, while mating significantly reduces lifespan and induces significant fat loss. However, all previous studies of the links between longevity and fat storage used worms with limited reproduction. While *daf-2* (Insulin signaling) and germlineless *glp-1* mutants are long-lived and store more fat, dietary restricted worms, such as *eat-2*, are long-lived but have less overall fat (Brooks et al., 2009; O’Rourke et al., 2009; Bar et al., 2016), making the relationship between fat levels and longevity less clear. Therefore, examining the relationship between fat metabolism and lifespan regulation in mated worms could provide new insights.

**FIGURE 1:**
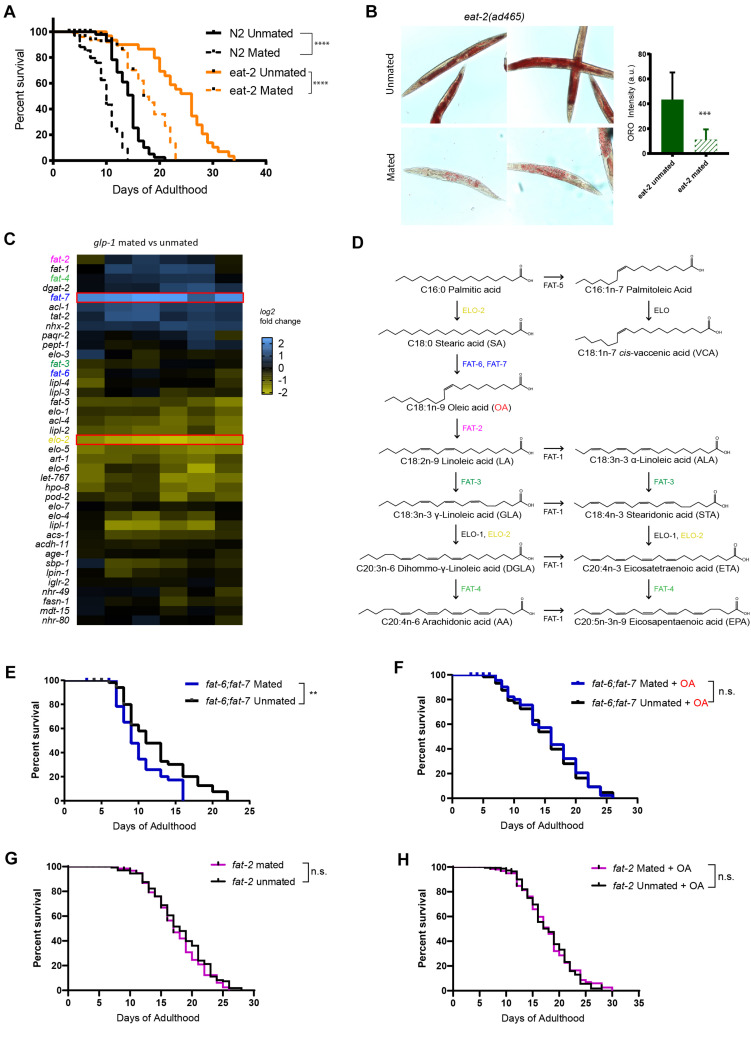
Endogenous oleic acid protects worms from mating-induced death. *t*-test, *p < 0.05, **p < 0.01, ***p < 0.001, ****p < 0.0001 for all graphs. For all the lifespan assays performed in this study, Kaplan–Meier analysis with log-rank (Mantel–Cox) test was used to determine statistical significance (lifespan of mated worms was always compared to unmated worms of the same genotype/treatment unless stated otherwise). Error bars represent SEM unless noted. n.s., not significant. a.u., arbitrary units. **(A)** Mated worms under dietary restriction live longer than those *ad libitum* despite extreme depletion of fat. N2 Unmated: 14.0 ± 0.4 days, *n* = 50, N2 mated: 8.8 ± 0.4 days, *n* = 100, *p* < 0.0001; *eat-2(ad465)* unmated: 23.6 ± 1.1 days, *n* = 50, *eat-2(ad465)* mated: 15.1 ± 1.2 days, *n* = 100, *p* < 0.0001. **(B)** Mating induces significant fat loss in *eat-2(ad465)* hermaphrodites. Left: representative Oil Red O staining pictures; Right: quantification of Oil Red O staining. **(C)** Expression heatmap of genes related to fatty acid biosynthesis in mated *C. elegans glp-1(e2141)* hermaphrodites. **(D)** Pathway of *de novo* fatty acid synthesis in *C. elegans*, including molecular structure of each fatty acid. **(E,F)** Mutants that cannot synthesize oleic acid have dramatic lifespan loss after mating, and oleic acid supplementation fully rescues the mating-induced lifespan decrease *fat-6;fat-7* unmated: 12.3 ± 0.7 days, *n* = 60; *fat-6;fat-7* mated: 9.6 ± 0.4 days, *n* = 100, *p* = 0.0059; *fat-6;fat-7* unmated + oleic acid: 15.1 ± 0.8 days, *n* = 60; *fat-6;fat-7* mated + oleic acid: 15.5 ± 0.8 days, *n* = 100, *p* = 0.7648. **(G,H)** Mutants that contain excess endogenous oleic acid are protected from lifespan loss after mating. *fat-2* unmated: 18.1 ± 0.4 days, *n* = 175; *fat-2* mated: 17.1 ± 0.4 days, *n* = 193, *p* = 0.2178; *fat-2* unmated + oleic acid: 18.0 ± 0.4 days, *n* = 173; *fat-2* mated + oleic acid: 18.0 ± 0.4 days, *n* = 215, *p* = 0.7598.

We previously showed that mating shortens lifespan and induces significant fat loss in both wild-type worms and longevity mutants, including those with excessive fat accumulation such as *daf-2* and *glp-1* (Supplementary Figures 1A-C, Shi and Murphy, 2014). We wondered whether the lifespan of longevity mutants with reduced fat storage would also be affected by mating. Dietary restriction decreases the overall fat content but robustly extends lifespan across organisms (Walker et al., 2005; Ruetenik and Barrientos, 2015; López-Otín et al., 2016). We tested the lifespan of mated worms under dietary restriction, using two methods of dietary restriction (Greer and Brunet, 2009). *eat-2* mutants have defects in pharyngeal pumping, thus consume less food than wild-type animals (Avery, 1993). Solid plate-based dietary restriction (sDR) takes the colony-forming unit of bacteria into account, using 1 × 10^11^ cfu/mL for *ad libitum* feeding and 1 × 10^8^ cfu/mL for dietary restriction (Greer and Brunet, 2009). We found that mating significantly decreases the lifespan of DR worms, just as it does in wild-type animals (Figure 1A and Supplementary Figure 1D). Similarly, mating also led to a significant fat loss in *eat-2* mutants, as evidenced by Oil Red O staining which stains the major fat stores, TAGs (O’Rourke et al., 2009; Figure 1B). Thus, the reduced level of overall fat storage prior to mating in DR-treated animals does not protect the worms from mating-induced death.

### Endogenous Oleic Acid Protects Worms Against Mating-Induced Death

To better understand how fat metabolism is altered after mating, we performed transcriptional analysis of mated vs. unmated *glp-1* hermaphrodites (Booth et al., 2020). *glp-1* mothers lack a germline to produce any eggs, which allowed us to disregard transcriptional changes in eggs, and instead focus on somatic changes. The lifespan of *glp-1* animals is decreased by mating and they lose over 40% of their fat stores (Shi and Murphy, 2014). Our transcriptional analysis revealed that genes with significant expressional changes in response to mating were enriched for *organic acid metabolic process* (*q*-value: 9.8e^–22^) and *lipid catabolic process* (*q*-value: 3e^–06^) (Supplementary Figure 2), which is consistent with the mating-induced fat loss phenotype. The lipid-regulating gene *fat-7* was significantly induced in mated worms, while *elo-2* was significantly downregulated (Figure 1C). Both of the genes are involved in polyunsaturated fatty acids (PUFA) synthesis: *fat-7* encodes a Δ9 desaturase enzyme that converts C18:0 stearic acid into C18:1n-9 oleic acid (OA), while *elo-2* encodes a key enzyme that regulates elongation of C16 and C18 fatty acids (Figure 1D). (There are three main types of fatty acids: saturated, monounsaturated, and polyunsaturated, which refers to fat molecules that have none, one, and more than one unsaturated carbon bond, respectively). ELO-2 functions in several different steps of the pathway, and loss of function of *elo-2* causes multiple defects (Kniazeva et al., 2003). Down-regulation of *elo-2* in mated worms likely contributes to mating-induced fat loss. By contrast, the up-regulation of *fat-7*, a fatty acid synthesis gene, in the mated worms suffering from significant fat loss is counterintuitive, suggesting that its upregulation is more likely to compensate for the severe mating-induced fat loss. FAT-6 and FAT-7 are required to generate oleic acid from its direct upstream precursor stearic acid (Figure 1D), and act in a compensatory mechanism in single mutants (Brock et al., 2006). *fat-6;fat-7* double mutants cannot endogenously synthesize oleic acid and downstream PUFAs, and instead rely solely on fatty acids provided by the bacterial diet (Brooks et al., 2009); OA is almost non-existent in the common lab bacteria, OP50 (Deline et al., 2013). *fat-2* encodes the desaturase enzyme that converts oleic acid to its direct downstream product, linoleic acid (Figure 1D). In *fat-2* worms, oleic acid accumulates to almost a quarter of all fat, which is nearly 15-fold above the 1.7% found in wild-type animals (Watts and Browse, 2002). Therefore, these two mutants, *fat-6;fat-7* and *fat-2*, represent the effects of lack of OA and accumulation of OA, respectively.

To understand the importance of endogenous oleic acid in mating-induced death, we compared these mutants’ mated and unmated lifespans. *fat-6;fat-7* mutants displayed many visible defects, such as slow growth, low brood size, and decreased body size, and have a short lifespan relative to wild-type worms (Brock et al., 2007). Mated *fat-6;fat-7* worms lived shorter than unmated *fat-6;fat-7* worms (Figure 1E), indicating that mating-induced death does not require the presence of OA. Supplementation with oleic acid greatly increased the lifespans of mated *fat-6;fat-7* worms, eliminating the difference between mated and unmated animals (Figure 1F), suggesting that the addition of exogenous OA both increases lifespan and eliminates mating-induced lifespan reduction.

By contrast with *fat-6;fat-7* mutants, *fat-2* mutants, which accumulate oleic acid, were both long-lived prior to mating (15% increase relative to unmated N2; *p* = 0.0002; pooled results from three biological replicates, Supplementary Figure 3A and Supplementary Table 1) and resistant to mating-induced lifespan reduction (Figure 1G, *p* = 0.2187). Further supplementation of oleic acid had no effect on *fat-2* lifespan (Figure 1H, *p* = 0.7598), suggesting that presence of endogenous oleic acid grants *fat-2* mutants protection from mating-induced death.

### OA Specifically Increases Mated Lifespan

**FIGURE 2:**
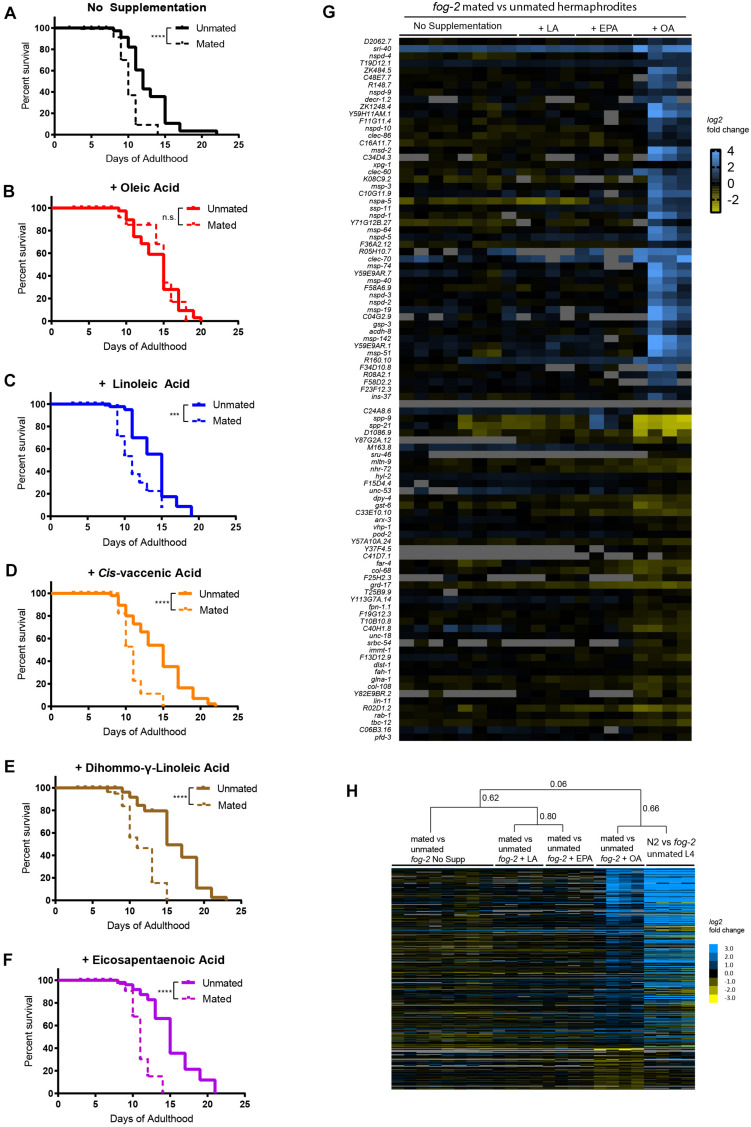
Oleic acid supplementation rescues mating-induced death in wild-type *C. elegans* mothers. **(A–F)** Only oleic acid supplementation rescues lifespan loss induced by mating. N2 unmated: 12.7 ± 0.6 days, *n* = 60; mated: 9.4 ± 0.4 days, *n* = 100, *p* < 0.0001; unmated + OA: 14.2 ± 0.5 days, *n* = 60; mated + OA: 13.3 ± 1.2 days, *n* = 100, *p* = 0.8344; unmated + LA: 14.3 ± 0.6 days, *n* = 60; mated + LA: 10.2 ± 0.4 days, *n* = 100, *p* = 0.0007; unmated + VCA: 14.3 ± 0.6 days, *n* = 60; mated + VCA: 10.5 ± 0.3 days, *n* = 100, *p* < 0.0001; unmated + DGLA: 15.9 ± 0.6 days, *n* = 60; mated + DGLA: 9.8 ± 0.5 days, *n* = 100, *p* < 0.0001; unmated + EPA: 15.2 ± 0.5, *n* = 60; mated + EPA: 10.3 ± 0.4, *n* = 100, *p* < 0.0001. **(G)** Heatmap of top 50 genes that were significantly differentially expressed upon mating with oleic acid supplementation identified by two-class SAM analysis (Tusher et al., 2001). **(H)** Heatmap of differentially expressed genes identified by two-class SAM analysis comparing the transcriptomes of mated vs. unmated worms with oleic acid supplementation to all other conditions. The same list of genes was retrieved from published data (N2 vs. *fog-2* unmated L4 hermaphrodites, Shi et al., 2019) and their heatmap were added on the right to show striking similarity. Hierarchical clustering of the arrays were performed by Cluster 3.0 (de Hoon et al., 2004) using centroid linkage method. Correlation coefficients are listed next to the corresponding branches/nodes.

Since excess endogenous oleic acid (via mutants) protects animals against mating-induced death, we wondered if supplementing mated worms with exogenous oleic acid could also mitigate mating-induced death. Therefore, we supplemented mated wild-type (N2) hermaphrodites with oleic acid or with fatty acids (FA) in the FA synthesis pathway to determine the specificity of fatty acid supplementation. Oleic acid (OA), linoleic acid (LA), *cis*-vaccenic acid (VCA), dihommo-γ-linoleic acid (DGLA), and eicosapentaenoic acid (EPA) were added to the agar media of mated and unmated wild-type *C. elegans* hermaphrodites. Consistent with a previous finding that dietary supplementation of oleic acid, palmitoleic acid, or *cis*-vaccenic acid extends the lifespan of unmated hermaphrodites (Han et al., 2017), the addition of each fatty acid caused a slight increase in the lifespan of unmated wild-type hermaphrodites (Figures 2A–F and Supplementary Table 1, lifespan increase of individual fatty acid supplementation: OA, 12%; LA, 11%; VCA, 11%; DGLA, 17%; EPA, 16%), demonstrating that these fatty acids are successfully being taken up by the worms. Mated N2 worms without any fatty acid supplementation had a ∼30% shorter lifespan than unmated worms (Figures 1A, 2A). While oleic acid supplementation completely rescued the lifespan loss caused by mating (Figure 2B), supplementation with any other fatty acid failed to rescue mating-induced lifespan decrease (Figures 2C–F), suggesting that oleic acid is specifically required for post-mating lifespan regulation. We supplemented the plates with various concentrations of oleic acid (OA) and found that OA showed a positive lifespan effect at low concentration (0.8 mM; 10% increase, *p*-value < 0.001) and completely inhibited mating-induced death at higher concentration (2 mM; 30% increase, *p* < 0.001) (Supplementary Figure 3B). While 2 mM is likely to achieve supraphysiological levels of OA, Han et al. (2017) showed that 0.8 mM fatty acid supplementation leads to a 2–3-fold increase in the corresponding FA in the worm.

To understand why only oleic acid is able to rescue mating-induced death, we compared the transcriptomes of mated vs. unmated hermaphrodites raised on normal agar media and on media with oleic acid, linoleic acid, and eicosapentaenoic acid supplementation. In all four conditions, mating led to dramatic transcriptional changes in hermaphrodites (Supplementary Table 2; one-class SAM). Principal component analysis separated mating-induced transcriptome changes based on the presence/absence of exogenous fatty acids supplementation (Supplementary Figure 4A), and to a lesser degree the specific type of fatty acid added (Supplementary Figure 4B). Similarly, supplementation of fatty acids accounted for the vast majority of difference in molecular functions, biological processes, cellular components, and transcription factors underlying mating-induced transcriptional changes revealed by Gene Ontology Enrichment analysis (Supplementary Figure 5), among which glycoprotein and peptidoglycan metabolic/catabolic processes (examples include lysozyme genes *lys-4*, *lys-5*, *lys-6*, Supplementary Figure 5B and Supplementary Table 3), and “pseudopodium” (mostly major sperm protein related genes, Supplementary Figure 5C and Supplementary Table 3) were enriched for genes that are up-regulated after mating with oleic acid supplementation.

To better understand how oleic acid specifically protects the worms from mating-induced death, we next performed two-class SAM analysis of the mated vs. unmated transcriptomes, and identified genes that were significantly differentially expressed upon mating *only* with oleic acid supplementation (FDR = 5%, *q* < 5.92%; Supplementary Table 4 and Figures 2G,H). In addition to genes encoding major sperm proteins and lysozymes as previously identified using one-class SAM analysis, we found several longevity genes and genes responsible for lipid metabolism/fatty acid storage that were specifically regulated in mated worms in the presence of oleic acid, including the serpentine receptor *sri-40*, which has recently been shown to regulate hermaphrodite lifespan both with and without males (Booth et al., 2020). Genes that regulate mitochondrial fatty acid beta oxidation, such as *acdh-8*, *decr-1.2*, and *decr-1.3*, were specifically up-regulated in mated worms with oleic acid supplementation, suggesting that oleic acid’s unique ability to modulate lipid homeostasis might contribute to its specificity in regulating the longevity of mated worms. Finally, *ins-37*, which encodes an insulin receptor antagonist (Zheng et al., 2018), was specifically up-regulated in oleic acid treated mated worms. We previously showed that self-sperm-induced overexpression of *ins-37* protects hermaphrodites from short-term mating (Shi et al., 2019). Indeed, the oleic acid-specific mating-induced up-regulated gene expression profile resembles the transcriptional pattern of young hermaphrodites with self-sperm (Figure 2H; Pearson correlation = 0.66; Shi et al., 2019). The striking similarity in transcriptional profiles between these two conditions suggests that oleic acid utilizes a similar pathway to that of worms that still contain their own sperm (Booth et al., 2019; Shi et al., 2019), to protect against mating-induced death, to live long. Therefore, oleic acid-mediated sustained up-regulation of *ins-37* may provide long-term protection from mating-induced death.

### Oleic Acid Supplementation Restores Lifespan and Fat Loss in Mated Worms Without Affecting Reproductive Health

**FIGURE 3:**
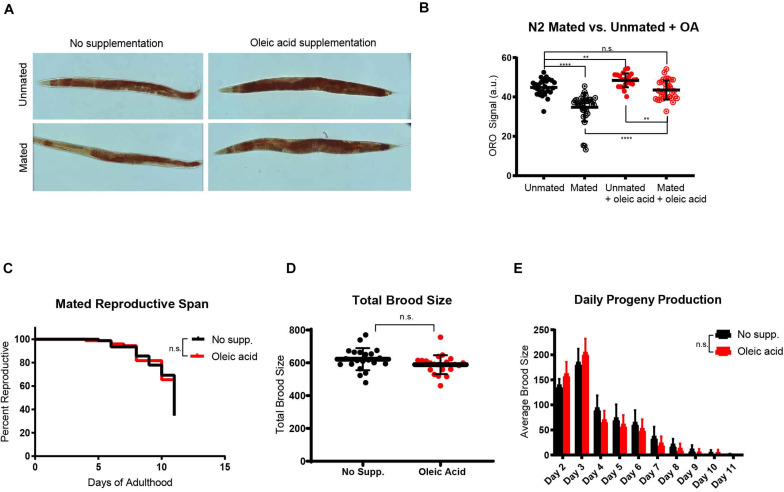
Oleic acid supplementation restores fat storage without changing the reproduction of *C. elegans* mothers. **(A)** Dietary supplementation of oleic acid restores overall fat storage in wild-type hermaphrodites. Representative pictures of Oil red O fat staining are shown. **(B)** Quantification of Oil red O fat staining. Error bars: SD. *t*-test, **p < 0.01, ****p < 0.0001, n.s., not significant. **(C)** The reproductive span of mated worms is not affected by oleic acid supplementation. **(D)** The total brood size of mated worms is not affected by oleic acid supplementation. **(E)** Daily progeny productions of mated worms with and without oleic acid supplementation are not statistically different.

Because lifespan, reproduction, and fat storage are closely coupled (Hansen et al., 2013), with fat acting at the nexus of longevity-reproduction trade off, we next checked the overall fat storage in wild-type mated and unmated worms in the presence and absence of exogenous oleic acid. Upon mating, animals normally lose a significant fraction of their somatic fat stores (Figures 3A,B; Shi and Murphy, 2014), a shift that is necessary for the normal development of progeny (Heimbucher et al., 2020). We found that oleic acid supplementation fully rescued mating-induced somatic fat loss in mated worms, and also increased fat storage in unmated worms by ∼10% (Figures 3A,B). However, neither progeny production rates nor total brood sizes are reduced upon OA treatment (Figures 3C–E) – that is, animals supplemented with OA are able to produce progeny without any trade-off in lifespan. These results suggest that oleic acid supplementation allows animals to reproduce fully without any loss of lifespan. Therefore, OA is critical for the restoration of longevity that is lost upon mating and progeny production, solving the longevity trade-off that reproduction normally costs.

### Lifespan Rescue Through Oleic Acid Supplementation Is Conserved

**FIGURE 4:**
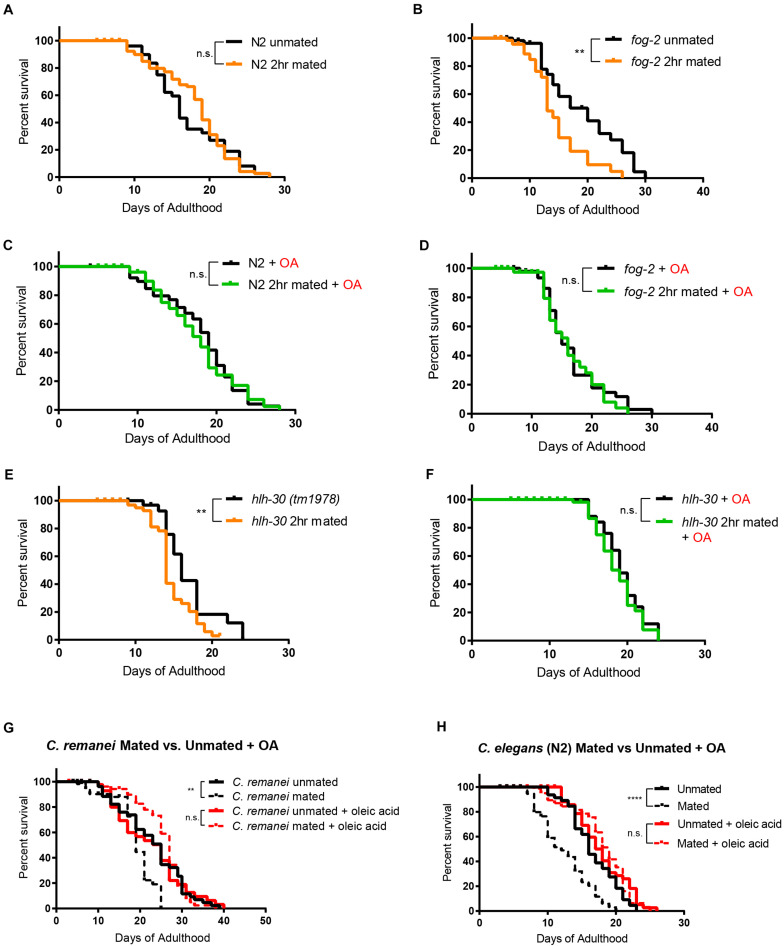
Oleic acid-specific mating-induced death rescue is conserved. Kaplan-Meier analysis with log-rank (Mantel-Cox) test was used to determine statistical significance for all the lifespan assays (lifespan of mated worms was always compared to unmated worms of the same genotype/treatment). **p < 0.01, ****p < 0.0001. **(A–F)** Oleic acid protects worms from short-term mating-induced death. **(A)** Unmated N2: 16.7 ± 0.8 days, *n* = 60; 2 h mated: 17.8 ± 0.5 days, *n* = 100, *p* = 0.5900. **(B)** Unmated *fog-2*: 18.2 ± 1.4 days, *n* = 60; *fog-2* 2 h mated: 14.0 ± 1.0 days, *n* = 100, *p* = 0.0044. **(C)** Unmated N2 + oleic acid: 17.4 ± 0.7 days, *n* = 60; 2 h mated + oleic acid: 17.7 ± 0.6 days, *n* = 100, *p* = 0.7676. **(D)** Unmated *fog-2* + oleic acid: 16.4 ± 0.8 days, *n* = 60; *fog-2* 2 h mated + oleic acid: 16.2 ± 0.9 days, *n* = 100, *p* = 0.5910. **(E)** Unmated *hlh-30*: 16.6 days ± 0.8 days, *n* = 60; *hlh-30* 2 h mated: 14.3 ± 0.4 days, *n* = 100, *p* = 0.0117. **(F)** Unmated *hlh-30* + oleic acid: 19.4 ± 0.5 days, *n* = 60; *hlh-30* 2 h mated + oleic acid: 18.8 ± 0.4 days, *n* = 100, *p* = 0.4357. **(G)** Mated *C. remanei* mothers’ lifespans are also rescued by oleic acid supplementation. Unmated: 22.6 ± 1.2 days, *n* = 60; mated: 18.2 ± 0.8 days, *n* = 60, *p* = 0.0029; unmated + oleic acid: 21.9 ± 1.4 days, *n* = 60; mated + oleic acid: 25.3 ± 0.9 days, *n* = 60, *p* = 0.3266. **(H)** Lifespans of unmated and mated N2 hermaphrodites with and without oleic acid supplementation. Unmated: 16.6 ± 0.5 days, *n* = 49; mated: 11.8 ± 0.5 days, *n* = 80, *p* < 0.0001; unmated + oleic acid: 17.9 ± 0.6 days, *n* = 50; mated + oleic acid: 18.1 ± 0.6 days, *n* = 80, *p* = 0.9466.

*Caenorhabditis elegans* hermaphrodites self-reproduce through internal self-fertilization with their own sperm (“self-sperm”) (Ward and Carrel, 1979). The presence of self-sperm protects the hermaphrodites from mating-induced death (Booth et al., 2019; Shi et al., 2019). We next wondered what role self-sperm-mediated protection might play in OA-mediated lifespan rescue. Previously, we found that self-sperm protects and slows down mating-induced death in *C. elegans* hermaphrodites through nuclear localization of HLH-30, the *C. elegans* ortholog of the mammalian transcription factor EB (TFEB), a conserved master regulator of autophagy and key pro-longevity regulator (Lapierre et al., 2013; Shi et al., 2019). We first tested *C. elegans* hermaphrodites with no self-sperm. The protective effect of OA was confirmed in short-term mating of N2 hermaphrodites for 2 h on Day 3 of adulthood when self-sperm is still present (Figure 4A); 2 h of mating is sufficient to induce a significant lifespan reduction in worms who lack self-sperm and therefore also lack the protective antagonist insulin (INS-37). Specifically, *fog-2* (feminization of germline) mutants lack self-sperm (Schedl and Kimble, 1988; Figure 4B) and are short-lived after mating. Oleic acid supplementation had no effect on lifespan on either unmated or 2 h-mated wild-type hermaphrodites that had self-sperm (Figure 4C), but oleic acid supplementation fully rescued the lifespan reduction of mated *fog-2* (self-spermless) mutants (Figure 4D). This conserved effect was further validated in *hlh-30* mutants that also suffer from 2 h-mating-induced death even in the presence of self-sperm (Figure 4E); oleic acid supplementation rescued *hlh-30’s* mated lifespan decrease, as well (Figure 4F), suggesting that oleic acid may act downstream of HLH-30/TFEB to regulate lifespan.

Finally, we wondered if oleic acid supplementation is specific for hermaphrodites, or whether it can also rescue the early death of mated gonochoristic species females. We showed previously that *C. remanei* females also have significantly reduced lifespan after mating with *C. remanei* males (Shi and Murphy, 2014). Remarkably, mated females supplemented with oleic acid lived as long as unmated females with and without supplementation (Figure 4G), just as in the case of *C. elegans* wild-type hermaphrodites (Figure 4H). These results suggest that lifespan rescue through oleic acid supplementation is evolutionarily conserved in mated *Caenorhabditis* mothers.

## Discussion

The trade-off between reproduction and lifespan has been observed in a variety of animals (Partridge et al., 2005; Hansen et al., 2013). Fat, as the major means of storing energy, has been shown to be closely involved in both reproduction and lifespan regulation. The same inverse relationship also exists between reproduction and fat storage (Hansen et al., 2013; Lynn et al., 2015; Heimbucher et al., 2020). Germline-less *glp-1* worms upregulate the Δ9 desaturase enzyme FAT-6/SCD1 (Stearoyl-CoA Desaturase 1) (Goudeau et al., 2011) and shift the lipid profile to increase somatic maintenance and longevity (Ratnappan et al., 2014), while deficiency of the H3K4me3 methyltransferase (*ash-2*, *set-2*) upregulates Δ9 desaturases FAT-5 and FAT-7, leading to the accumulation of mono-unsaturated fatty acids and subsequent lifespan extension (Han et al., 2017).

**FIGURE 5:**
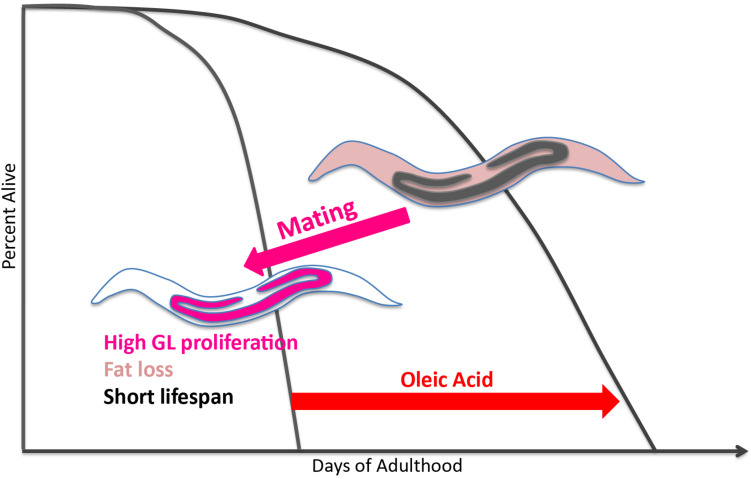
Model of oleic acid-mediated lifespan restoration in mated worms. Mating leads to germline hyperactivity, fat loss, and shortened lifespan in *Caenorhabditis* mothers. Oleic acid addition (whether endogenously or exogenously) is able to restore the lifespan decrease caused by mating.

By contrast, mated hermaphrodites demonstrate the inverse relationship between reproduction, longevity, and fat storage: mating increases progeny production by 2–3 fold, reduces fat stores, and significantly shortens lifespan compared to unmated hermaphrodites. Here, we have shown that oleic acid, a product of the Δ9 desaturase enzyme FAT-7 – whether through its endogenous accumulation by loss of FAT-2 activity or through exogenous supplementation – can prevent mating-induced death (Figure 5). Furthermore, the lifespan-rescuing effect of oleic acid is evolutionarily conserved in self-spermless hermaphrodites and females of gonochoristic *Caenorhabditis* species. Dietary supplementation of oleic acid as well as a few other monounsaturated fatty acids (MUFAs), including palmitoleic and *cis*-vaccenic acid can extend the lifespan of unmated hermaphrodites (Han et al., 2017). However, supplementing other fatty acids is not able to ameliorate the mating-induced death. Additionally, oleic acid supplementation leads to a much larger lifespan extension in mated worms compared to unmated hermaphrodites (Supplementary Figure 6). Taken together, our results demonstrate the specificity of oleic acid in regulating post-mating lifespan, and suggest that it is slightly different from H3K4me3 methyltransferase-mediated transgenerational inheritance of longevity.

Our analysis of mated animals with increased reproduction revealed that overall (total) fat levels play no role in the lifespan regulation of mated worms; that is, mating reduces the lifespan of longevity mutants, whether they exhibit increased or decreased fat levels prior to mating. Our transcriptome analysis of mated and unmated worms revealed mating-induced upregulation of regulators of fatty acid metabolism. Only oleic acid treatment can rescue fat loss induced by mating and lifespan reduction induced by mating, without affecting reproduction. Our additional transcriptome analysis comparing mated vs. unmated worms on media containing different types of fatty acids revealed that only oleic acid supplementation induces self-sperm-like protective transcriptional pattern in mated worms. Specifically, the insulin-like peptide antagonist, INS-37, which we previously found to be expressed at high levels in worms that still contain self-sperm, and which prevents male-sperm mating-induced death (Shi et al., 2019), is expressed at higher levels in oleic acid-treated worms.

Our results suggest that oleic acid depletion in the soma is the ultimate downstream cost of reproduction. Oleic acid might affect the soma through maintenance of membrane fluidity (stearic acid/oleic acid ratio) (Funari et al., 2003) or because of general lipid storage. It is notable that other lipids in the pathway were not able to rescue mating-induced death, suggesting that this specific fatty acid has a unique role in somatic maintenance and growth. The specificity of oleic acid has also been reported in mediating the age-dependent somatic depletion of fat (Asdf) (Lynn et al., 2015), as oleic acid is the only fatty acid that is able to rescue the Asdf phenotype. Even though the Asdf phenotype was examined in unmated worms and under oxidative stress, which differs from the mating-induced death in this study, both phenotypes involve the interaction between the soma and germline. The fact that the specificity of oleic acid is observed in both studies further increases the likelihood of oleic acid as the possible link between germline/reproduction and somatic maintenance/lifespan. Oleic acid, more than other fatty acids that did not affect longevity, regulates the expression of several genes involved in fatty acid storage.

In addition to dietary supplementation, we also show that endogenous oleic acid is beneficial to mated worms. Genetically blocking FAT-2, the desaturase enzyme that converts oleic acid to linoleic acid, increases endogenous oleic acid levels (Watts and Browse, 2002) as well as the level of total triglyceride (TAG) (Horikawa and Sakamoto, 2010), which we find protects the worms from mating-induced death. FAT-6 and FAT-7 are the stearoyl CoA desaturases (SCD) that convert stearic acid to oleic acid. Interestingly, *fat-6* and *fat-7* are up-regulated in long-lived mutants such as *daf-2* and *glp-1* (Murphy et al., 2003; Goudeau et al., 2011), indicating again that increased endogenous oleic acid is beneficial to worms. It is possible that oleic acid is the somatic energy source that extends lifespan in these mutants. FAT-6/FAT-7/SCD1 desaturase enzyme activity is highly conserved. In mammals including humans, oleic acid is the prime substrate for triglyceride (Chu et al., 2006). Loss of SCD1, which synthesizes oleic acid, was shown to protect against adiposity by preventing the accumulation of triglyceride in mice (Ntambi et al., 2002), just as *fat-6;fat-7* mutant worms have significantly low levels of triglyceride storage (Watts and Browse, 2002). Furthermore, SCD1 is strongly upregulated in response to ovariectomy in mice (Paquette et al., 2008). The biological roles of oleic acid and desaturation are thus conserved in several aspects between *C. elegans* and humans. With regard to reproduction, oleic acid is a major determinant of plasma membrane fluidity in many cells, including follicular cells (Funari et al., 2003). In bovine oocytes, oleic acid plays a key role in membrane fluidity that counteracts the detrimental effects of exposure to saturated fatty acids (Aardema et al., 2011), and acts as a metabolic regulator of oxidative stress and cellular signaling in oocytes, early embryonic development, and female fertility (Fayezi et al., 2018).

Oleic acid is likely to be the linchpin of the reproductive and somatic energy balance. Mating-induced up-regulation of *fat-7*, which converts stearic acid to oleic acid, could be a response of the mated worms to prepare themselves with more endogenous oleic acid for dramatically increased progeny production. At the same time, the somatic reserve of oleic acid is depleted as indicated by the loss of fat storage to fuel the mating-induced elevated reproductive activity. In this case, mated worms prioritize oleic acid for reproduction, and the outcome is increased reproduction at the cost of lifespan. By contrast, in *daf-2* and *glp-1* mutants with reduced or no reproductive activity, more oleic acid accumulates in the somatic tissues and may be utilized for somatic maintenance, leading to an extension of lifespan. Therefore, oleic acid is likely to be the limiting energy source that reproduction and somatic maintenance compete for. Our results suggest that oleic acid depletion is a prime example of the cost of reproduction. Excitingly, it is one that appears to be reversible through dietary supplementation of oleic acid. Oleic acid, along with the evolutionarily conserved enzymes in the pathway, might be therapeutic targets that could help humans improve reproductive and somatic health at the same time without having to sacrifice one for the other.

## Materials and Methods

### Strains and Maintenance

All worm strains used in this study are listed below. All strains were maintained on Nematode Growth Media (NGM) plates at 20°C according to the standard procedure (Brenner, 1974).

**Table:** 

Strain	Genotype	Source
N2	Wild-type	CGC
CB4108	*fog-2(q71) V*	CGC
CB4037	*glp-1(e2141) III*	CGC
RB969	*fat-2(ok873) IV*	CGC
BX156	*fat-6(tm331) IV;fat-7(wa36)V*	CGC
JIN1375	*hlh-30(tm1978) IV*	CGC
PB4641	*Caenorhabditis remanei*	CGC

*CGC is the *Caenorhabditis* Genetics Center.*

### Mating Assays

All assays were performed at 20°C, and all worms were synchronized through bleaching adult worms with a mixture of NaOCl and KOH, leaving only the eggs.

#### Twenty-Four Hour Group Mating

Ten centimeter NGM plate was seeded with 300 μL of OP50 (Brenner, 1974) to create a ∼3 cm diameter of bacterial lawn. Hundred hermaphrodites and 300 males at late L4 were transferred onto the plate. The ratio of 1 hermaphrodite to 3 males was used to optimize mating. 24 h later, the hermaphrodites were transferred onto newly seeded plates without males. Complete mating means that males transfer enough sperm so that all progeny produced are cross-progeny (half males, half hermaphrodites). Incomplete mating means that males do not transfer enough sperm so that their sperm is used up and hermaphrodites later revert to using their own sperm, evidently seen from the ratio of male: hermaphrodite offspring. The progeny of the mated hermaphrodites was checked every day, and incompletely mated worms were censored.

For *C. remanei*, the same method was used for mating. Since males deposit copulatory plugs onto females’ vulva after mating, mated females without the plugs and unmated females with the plugs were both censored.

#### Two Hour Short-Term Mating

The same setup was used as group mating, but the mating was allowed for only 2 h. Hermaphrodites were transferred first onto seeded plates, and males were moved onto the plates later in an orderly and timely manner, so that each experimental group has exactly 2 h for mating.

### Lifespan Assay

Lifespan assays were performed using 3 cm NGM plates seeded with 25 μL of OP50. A maximum of 20 worms were present at each plate. For mated group, *n* = 100 while for unmated group, *n* > 50. Worms were transferred every day to freshly seeded plates during the reproductive period to eliminate confounding progeny, and every other day afterward. Worms were scored dead if they did not respond to repeated prods with a pick. The beginning of Day 1 of adulthood was defined as *t* = 0, and Kaplan–Meier survival analysis was performed with the log-rank (Mantel–Cox) method. Lifespan curves were generated using GraphPad Prism. Bagged (matricide), exploded, or lost worms were censored.

*fat-6;fat-7* mutants had suspended growth, so they were considered Day 1 adults when the egg-laying began.

### Reproductive Span and Brood Size Assay

Individual hermaphrodites after mating were transferred onto 3 cm NGM plates seeded with 25 μL of OP50 and moved to fresh plates daily until reproduction ceased for at least 2 days. The old plates were left at 20°C for 2 days to allow the offspring to grow into adults, which were manually counted for daily production and total brood size. At least 20 plates of individual worms were counted to account for individual variation. The last day of reproduction was marked as the last day of progeny production, evidenced by no worms on the old plates. When matricide occurred, the worm was censored from the experiment on that day. Kaplan–Meier analysis with log-rank (Mantel–Cox) method was performed to compare the reproductive spans. Reproductive span curves and brood size values were graphed using GraphPad Prism.

### Fatty Acid Supplementation

The fatty acid supplementation was adapted from the published protocol (Deline et al., 2013) with minor modifications from a more recent publication (Han et al., 2017). The detergent Tergitol (type NP-40, Sigma-Aldrich) was added to a final concentration of 0.001% in liquid NGM media, and an optimized concentration of 0.8 mM was supplemented for all fatty acids. For our purpose, fatty acids were in ethanol solution and were further dissolved in purified water by shaking for 30 min. The working stock was made fresh every time. The media was stirred for at least 5 min after the working stock was added. The fatty acids were deemed incompletely dissolved if a similar number of oil bubbles can be seen in the media with and without the detergent. Plates were kept inside a dark plastic box with no ventilation, but with sterile lab tissues that were replaced every day to absorb moisture. This method enabled the plates to remain without visible blackening of the fatty acid bubbles for a longer period. Control plates were made using the exact same procedure including the detergent and the ethanol, but not the fatty acid. Correct and consistent application of this procedure was essential for the replicability of the results.

### Oil Red O Staining and Quantification

Oil Red O staining was adapted from the published protocol to stain around 100 worms per condition (Wählby et al., 2014). Approximately 10 worms were imaged per slide with Nikon Ti. Oil Red O quantification was performed as published (O’Rourke et al., 2009). Briefly, colored images were split into RGB monochromatic images in Image J. The polygon selection tool was used to outline the boundary of each worm. The Oil Red O staining arbitrary unit (a.u.) was determined by mean gray value within the worm, calculated by the difference between the signals in Blue/Green and Red channel. T-test analysis was performed to compare the fat staining of different groups of worms. Individual intensity values were graphed using GraphPad Prism.

### Microarrays

Microarray data of mated and unmated *glp-1(e2141)* was retrieved from the previous publication (Booth et al., 2020). *glp-1(e2141)* hermaphrodites grown at the restrictive temperature (25°C) were mated on day 1 of adulthood for 24 h in a 2:1 male:hermaphrodite ratio. Two hundred hermaphrodites were collected on day 2/3 of adulthood for each biological replicate. In fatty acids supplementation experiments, eggs from bleached *fog-2(q71)* hermaphrodites were grown on NGM plates with various fatty acids supplementation at the final concentration of 1 mM. In mated groups, *fog-2* hermaphrodites were mated with young males for 24 h from day 1 of adulthood to day 2 of adulthood in the presence of fatty acid supplementation. About 200 hermaphrodites were collected on day 3 of adulthood for each biological replicate. RNA was extracted by the heat vortexing method. Two-color Agilent microarrays were used for expression analysis. Significantly differentially expressed gene sets were identified using SAM (Tusher et al., 2001). One class SAM was performed to identify genes that are significantly differentially expressed after mating in each fatty acid supplementation condition (Supplementary Figure 5). Two class SAM was performed to identify genes that are significantly differentially expressed after mating specific to oleic acid supplementation (Figures 2G,H). Principal component analysis was performed in R using prcomp function based on the genes identified by one class SAM (Supplementary Figure 4). Gene ontology enrichment analysis was performed using wormbase enrichment tools (Supplementary Figure 2B)^1^, and multi-query function of gProfiler (Supplementary Figure 5) (Raudvere et al., 2019).

Original microarray dataset can be found in PUMAdb:

Mated and unmated *glp-1(e2141)* hermaphrodites: https://puma.princeton.edu/cgi-bin/exptsets/review.pl?exptset_no=7345.

Mated and unmated *fog-2(q71)* hermaphrodites with different fatty acids supplementation: https://puma.princeton.edu/cgi-bin/exptsets/review.pl?exptset_no=7359.

## Data Availability Statement

The datasets presented in this study can be found in online repositories. The names of the repository/repositories and accession number(s) can be found in the article/Supplementary Material.

## Author Contributions

LSC and CTM designed the research. LSC, CS, JA, and SS performed the research and analyzed the data. LSC, CS, and CTM wrote the paper. All authors listed have made a substantial, direct and intellectual contribution to the work, and approved it for publication.

## Conflict of Interest

The authors declare that the research was conducted in the absence of any commercial or financial relationships that could be construed as a potential conflict of interest.

## Footnotes

1 https://wormbase.org/tools/enrichment/tea/tea.cgi

## Supplementary Material

Supplementary Figure 1 Mating causes dramatic fat loss and lifespan decrease in longevity mutants. **(A)** *daf-2(e1370)* longevity mutants are short-lived post-mating. Unmated *daf-2*: 37.0 ± 1.9 days, *n* = 47, mated *daf-2*: 19.4 ± 2.2 days, *n* = 60, *p* < 0.0001. **(B)** *glp-1(e2141)* mutants live shorter after mating as well. Unmated *glp-1*: 24.1 ± 1.3 days, *n* = 48, mated *glp-1*: 11.6 ± 0.5 days, *n* = 60, *p* < 0.0001. **(C)** Quantification of Oil red O fat staining of mated and unmated wild-type (N2), *daf-2*, and *glp-1* worms. **(D)** Mated worms under solid plate-based dietary restriction (sDR) live longer than those *ad libitum*. N2 Unmated: 14.0 ± 0.4 days, *n* = 50, N2 mated: 8.8 ± 0.4 days, *n* = 100, *p* < 0.0001; unmated + sDR: 23.7 ± 1.4 days, *n* = 50, mated + sDR: 14.1 ± 2.0 days, *n* = 100, *p* = 0.0042.

Supplementary Figure 2 Genome-wide transcriptional analysis of mated vs. unmated *glp-1* hermaphrodites. **(A)** Heatmap of whole transcriptome comparison between mated and unmated *glp-1(e2141)* hermaphrodites. **(B)** Gene ontology enrichment analysis of significantly differentially expressed genes.

Supplementary Figure 3 Both endogenous and exogenous oleic acid influence the lifespan of *C. elegans*. **(A)** Worms with excessive endogenous oleic acid are longer-lived. Lifespans: N2 unmated: 15.8 ± 0.4 days, *n* = 160; *fat-2* unmated: 18.1 ± 0.4 days, *n* = 173, *p* = 0.0002. **(B)** Dosage test of oleic acid supplementation. With improved fatty acid dissolution technique, 0.8 mM of supplementation was deemed sufficient for all later experiments. Unmated N2: 14.2 ± 0.5 days, *n* = 50; mated: 9.5 ± 0.3 days, *n* = 100; mated + 0.8 mM oleic acid: 12.2 ± 0.6 days, *n* = 100; mated + 2.0 mM oleic acid: 13.8 ± 0.7 days, *n* = 100.

Supplementary Figure 4 Transcriptomes of mated vs. unmated hermaphrodites are affected by fatty acids supplementation. **(A)** Principal component analysis of the transcriptomes of mated vs. unmated hermaphrodites raised on agar media with oleic acid (OA), linoleic acid (LA), eicosapentaenoic acid (EPA), and no supplementation (Ctrl). **(B)** Principal component analysis of the transcriptomes of mated vs. unmated hermaphrodites raised on agar media with oleic acid (OA), linoleic acid (LA), and eicosapentaenoic acid (EPA) supplementation.

Supplementary Figure 5 Gene Ontology analysis of transcriptomes of mated vs. unmated hermaphrodites with different fatty acids supplementation and control. Enrichment of molecular function **(A)**, biological process **(B)**, cellular component **(C)**, and transcription factor **(D)** were compared for all four conditions: worms raised on normal media (ctrl), on media with oleic acid (OA), linoleic acid (LA), and eicosapentaenoic acid (EPA) supplementation.

Supplementary Figure 6 Oleic acid supplementation increases the lifespan of mated worms more significantly. **(A)** Percent lifespan increase of the mated worms in the presence of oleic acid supplementation (30%; all genotypes included, each dot represents one lifespan replicate) is significantly higher than that of the unmated worms (5%), *p* = 0.0159, unpaired *t*-test. **(B)** Oleic acid supplementation does not affect the lifespans of *fat-2* mutants which have excessive endogenous oleic acid: only about 2% increase. The rest of the mated worms have an average of 40% lifespan increase in the presence of oleic acid supplementation compared to the 6% lifespan increase of the unmated worms, *p* = 0.0071, unpaired *t*-test. **(C)** Percent lifespan increase with the oleic acid treatment of each lifespan experiment with genotypes listed.

Supplementary Table 1 Lifespan Summary.

Supplementary Table 2 Mating-induced differentially expressed genes identified by one-class SAM analysis.

Supplementary Table 3 Gene Ontology Enrichment Analysis of genes in Supplementary Table 2.

Supplementary Table 4 Oleic acid-specific mating-induced differentially expressed genes identified by two-class SAM analysis.
