# Supplementary figures and images for: Oleic Acid Protects Caenorhabditis Mothers From Mating-Induced Death and the Cost of Reproduction
Source: Front Cell Dev Biol. 2021 Jun 11;9:690373. doi: 10.3389/fcell.2021.690373 (PMC8226236; doi:10.3389/fcell.2021.690373)

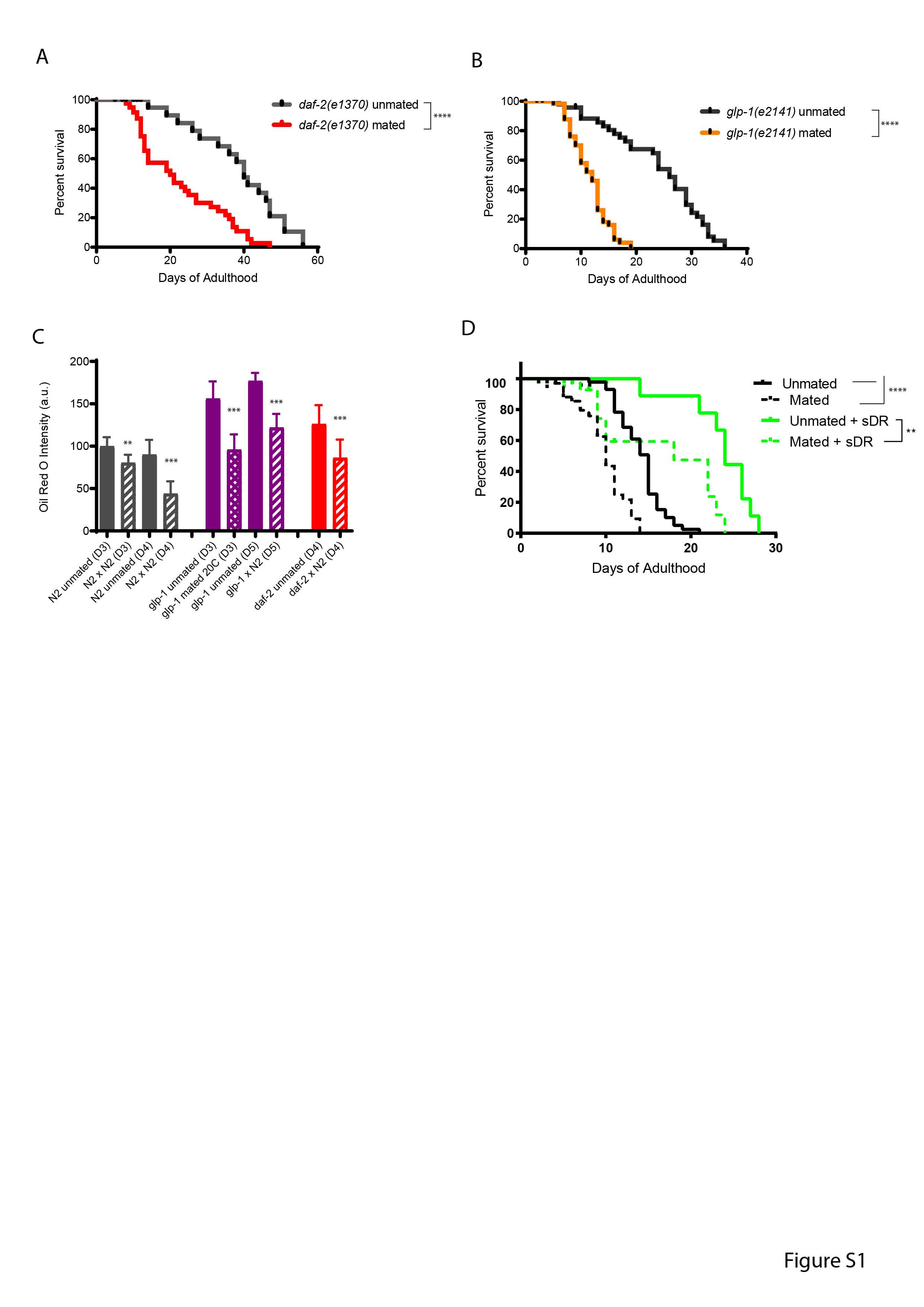

Supplement: Supplementary Figure 1 — Mating causes dramatic fat loss and lifespan decrease in longevity mutants. (A) daf-2(e1370) longevity mutants are short-lived post-mating. Unmated daf-2: 37.0 ± 1.9 days, n = 47, mated daf-2: 19.4 ± 2.2 days, n = 60, p < 0.0001. (B) glp-1(e2141) mutants live shorter after mating as well. Unmated glp-1: 24.1 ± 1.3 days, n = 48, mated glp-1: 11.6 ± 0.5 days, n = 60, p < 0.0001. (C) Quantification of Oil red O fat staining of mated and unmated wild-type (N2), daf-2, and glp-1 worms. (D) Mated worms under solid plate-based dietary restriction (sDR) live longer than those ad libitum. N2 Unmated: 14.0 ± 0.4 days, n = 50, N2 mated: 8.8 ± 0.4 days, n = 100, p < 0.0001; unmated + sDR: 23.7 ± 1.4 days, n = 50, mated + sDR: 14.1 ± 2.0 days, n = 100, p = 0.0042. [file Image_1.jpeg]

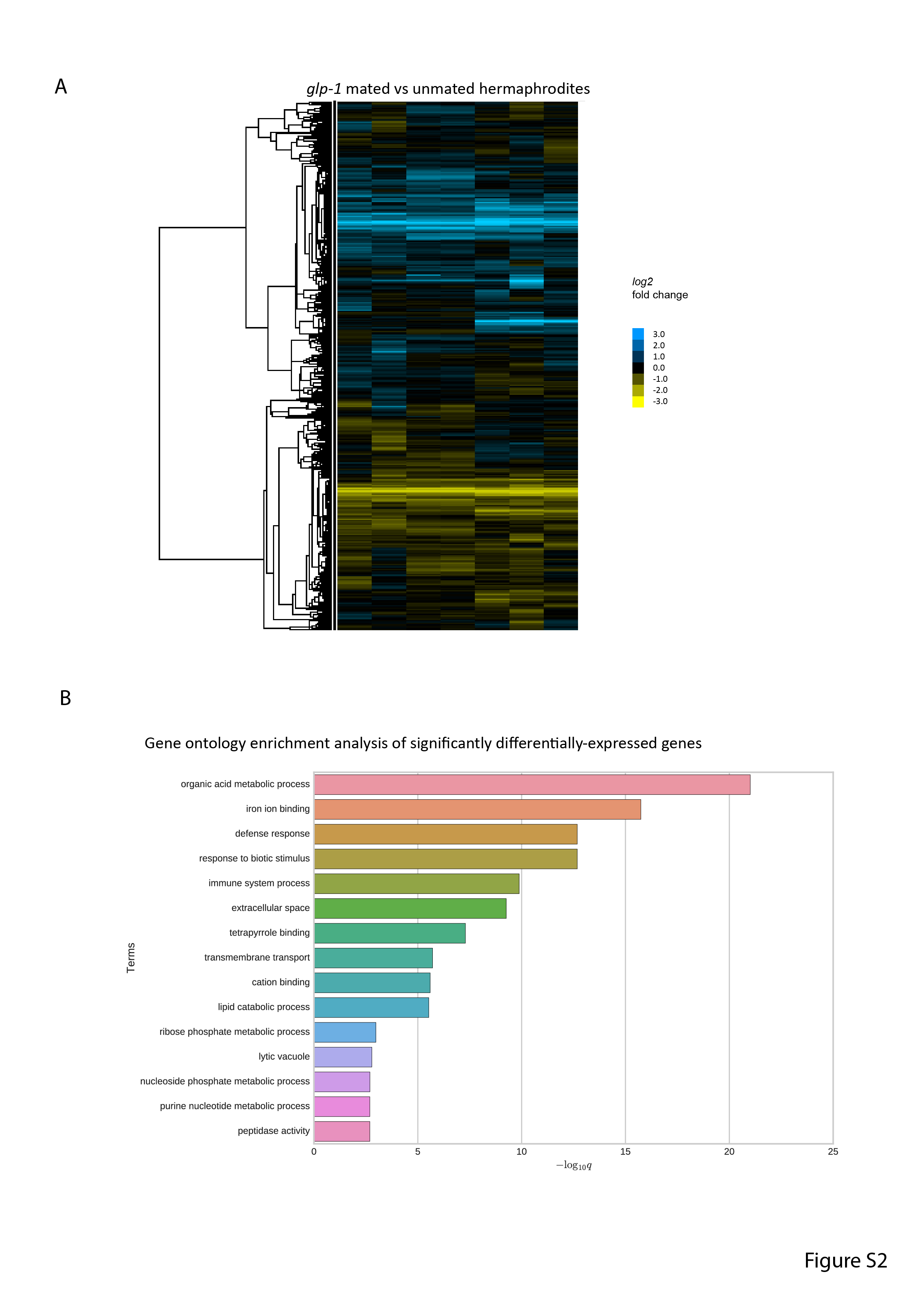

Supplement: Supplementary Figure 2 — Genome-wide transcriptional analysis of mated vs. unmated glp-1 hermaphrodites. (A) Heatmap of whole transcriptome comparison between mated and unmated glp-1(e2141) hermaphrodites. (B) Gene ontology enrichment analysis of significantly differentially expressed genes. [file Image_2.jpeg]

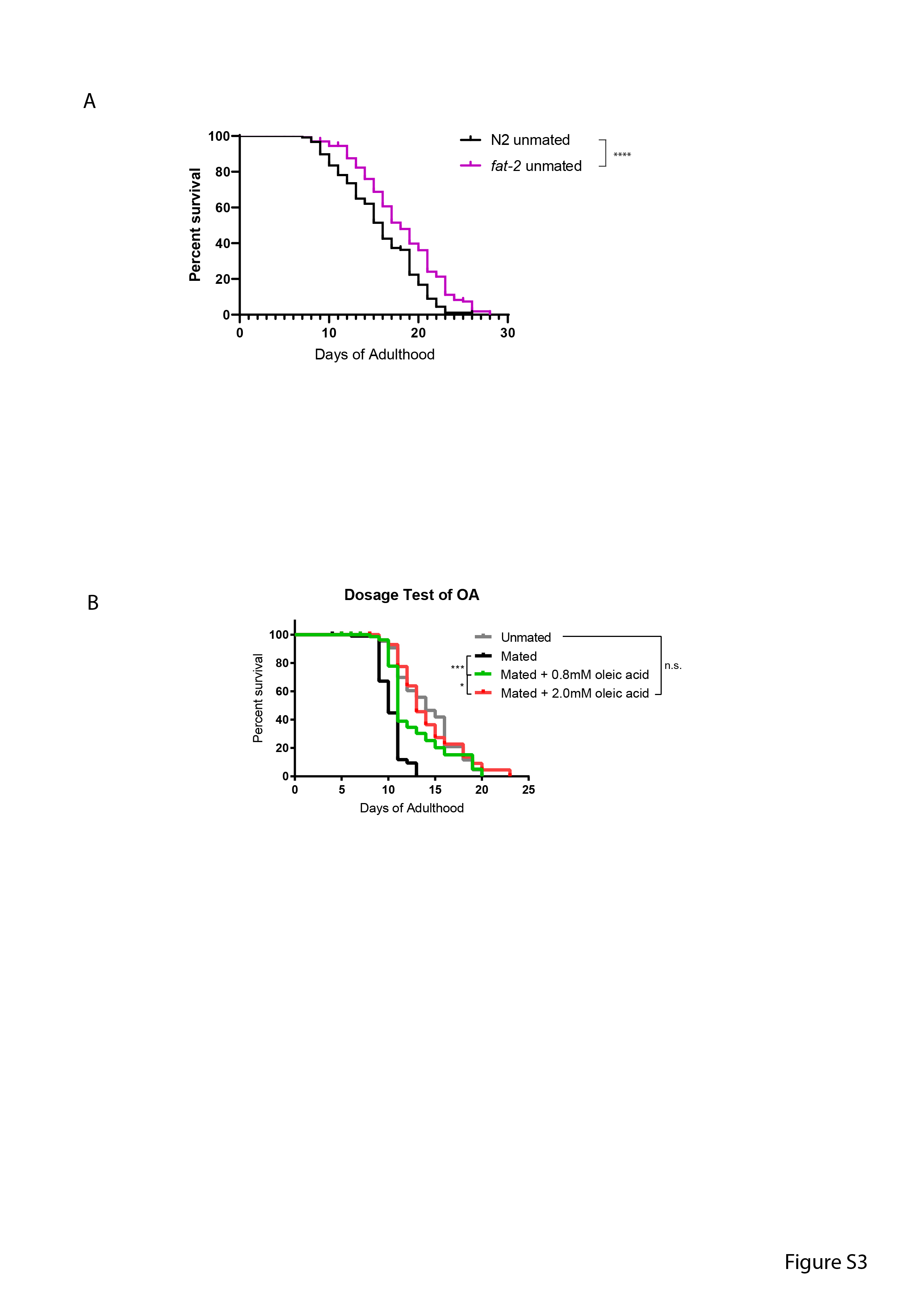

Supplement: Supplementary Figure 3 — Both endogenous and exogenous oleic acid influence the lifespan of C. elegans. (A) Worms with excessive endogenous oleic acid are longer-lived. Lifespans: N2 unmated: 15.8 ± 0.4 days, n = 160; fat-2 unmated: 18.1 ± 0.4 days, n = 173, p = 0.0002. (B) Dosage test of oleic acid supplementation. With improved fatty acid dissolution technique, 0.8 mM of supplementation was deemed sufficient for all later experiments. Unmated N2: 14.2 ± 0.5 days, n = 50; mated: 9.5 ± 0.3 days, n = 100; mated + 0.8 mM oleic acid: 12.2 ± 0.6 days, n = 100; mated + 2.0 mM oleic acid: 13.8 ± 0.7 days, n = 100. [file Image_3.jpeg]

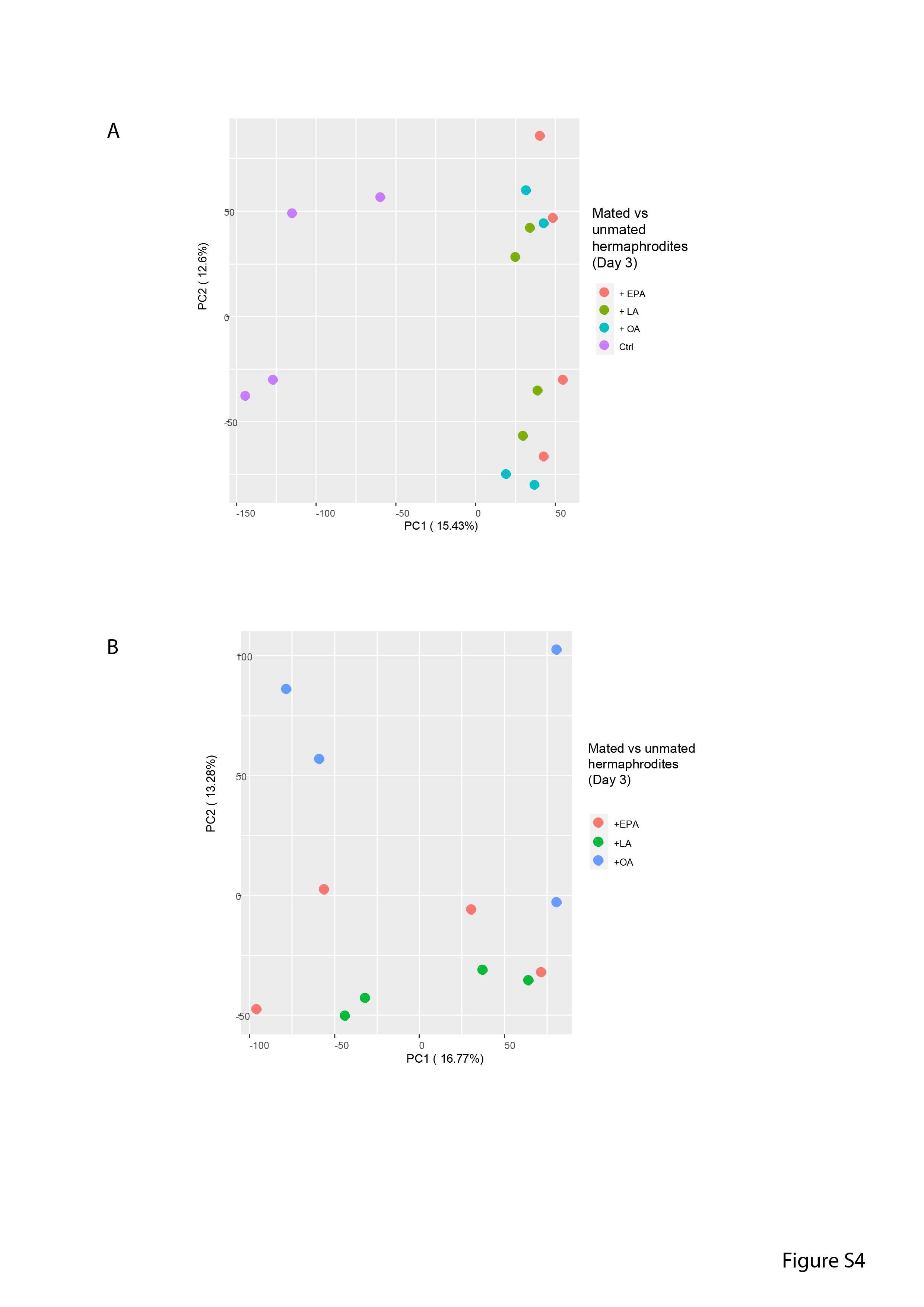

Supplement: Supplementary Figure 4 — Transcriptomes of mated vs. unmated hermaphrodites are affected by fatty acids supplementation. (A) Principal component analysis of the transcriptomes of mated vs. unmated hermaphrodites raised on agar media with oleic acid (OA), linoleic acid (LA), eicosapentaenoic acid (EPA), and no supplementation (Ctrl). (B) Principal component analysis of the transcriptomes of mated vs. unmated hermaphrodites raised on agar media with oleic acid (OA), linoleic acid (LA), and eicosapentaenoic acid (EPA) supplementation. [file Image_4.jpeg]

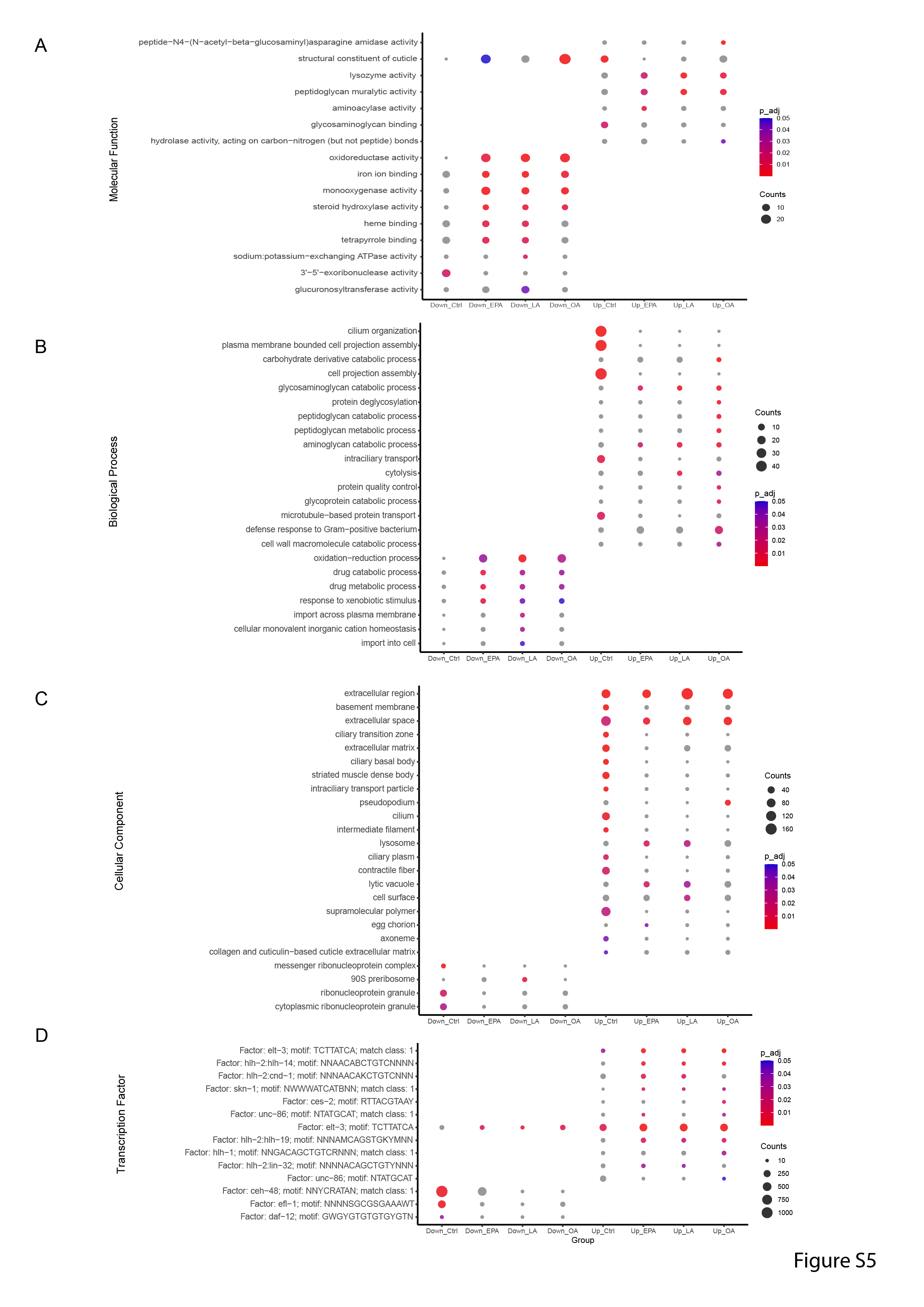

Supplement: Supplementary Figure 5 — Gene Ontology analysis of transcriptomes of mated vs. unmated hermaphrodites with different fatty acids supplementation and control. Enrichment of molecular function (A), biological process (B), cellular component (C), and transcription factor (D) were compared for all four conditions: worms raised on normal media (ctrl), on media with oleic acid (OA), linoleic acid (LA), and eicosapentaenoic acid (EPA) supplementation. [file Image_5.jpeg]

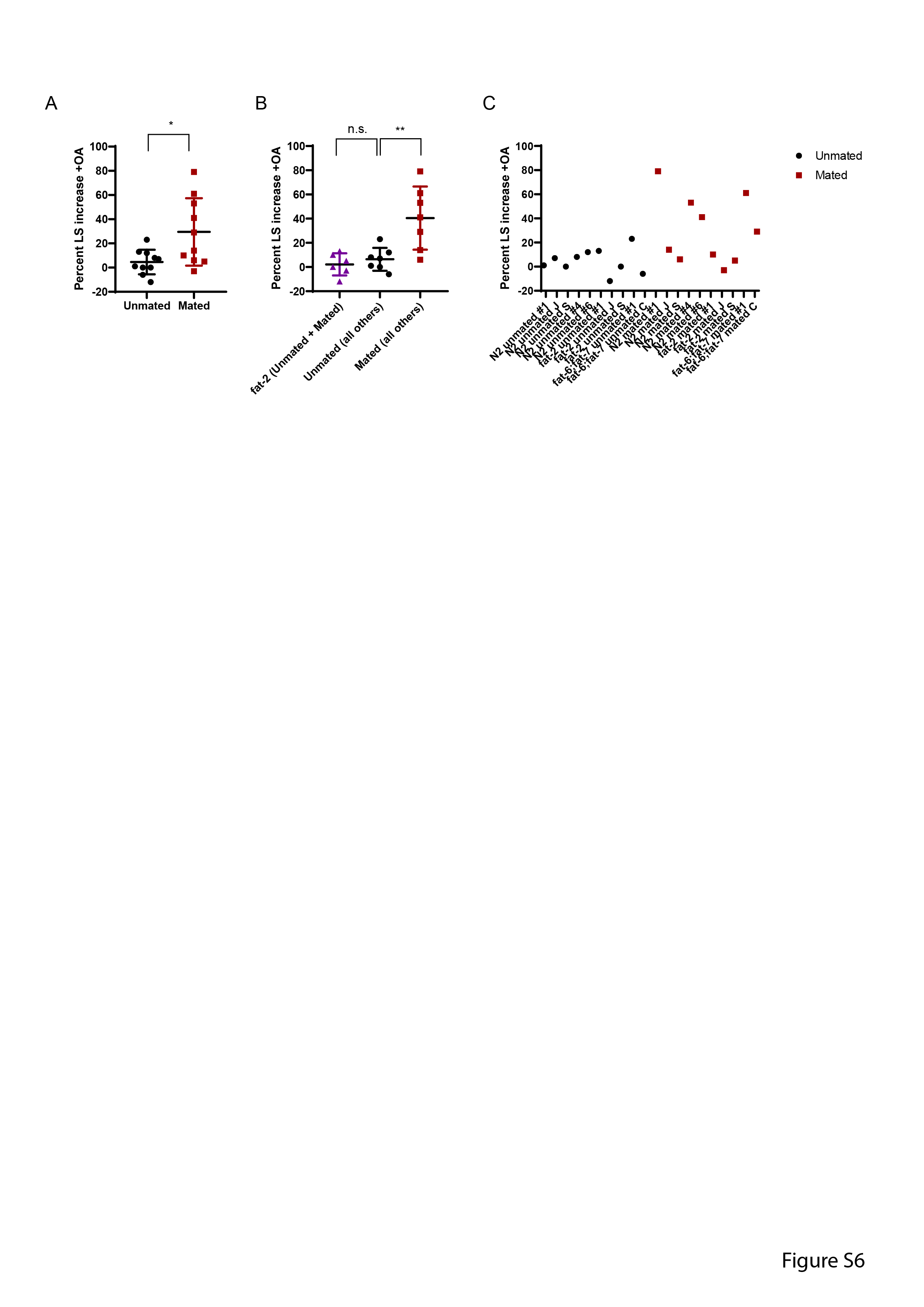

Supplement: Supplementary Figure 6 — Oleic acid supplementation increases the lifespan of mated worms more significantly. (A) Percent lifespan increase of the mated worms in the presence of oleic acid supplementation (30%; all genotypes included, each dot represents one lifespan replicate) is significantly higher than that of the unmated worms (5%), p = 0.0159, unpaired t-test. (B) Oleic acid supplementation does not affect the lifespans of fat-2 mutants which have excessive endogenous oleic acid: only about 2% increase. The rest of the mated worms have an average of 40% lifespan increase in the presence of oleic acid supplementation compared to the 6% lifespan increase of the unmated worms, p = 0.0071, unpaired t-test. (C) Percent lifespan increase with the oleic acid treatment of each lifespan experiment with genotypes listed. [file Image_6.jpeg]
