# Supplementary material for: Oleic Acid Protects Caenorhabditis Mothers From Mating-Induced Death and the Cost of Reproduction
Source: Front Cell Dev Biol. 2021 Jun 11;9:690373. doi: 10.3389/fcell.2021.690373 (PMC8226236; doi:10.3389/fcell.2021.690373)
Supplement: Supplementary Table 1 — Lifespan Summary. [file Table_1.DOCX]

**Lifespan assays summary**

| **Genotype/condition** | **Mean LS ± std. error** | **% change** | **p value** | **N** | **Fig** |
| --- | --- | --- | --- | --- | --- |
| **Figure 1** |  |  |  |  |  |
| N2 unmated | 14.0 ± 0.4 | -- | -- | 50 | Fig. 1A |
| N2 mated | 8.8 ± 0.4 | -37% | <0.0001 | 100 | Fig. 1A |
|  |  |  |  |  |  |
| eat-2(ad465) unmated | 23.6 ± 1.1 | -- | -- | 50 | Fig. 1A |
| eat-2(ad465) mated | 15.1 ± 1.2 | -36% | <0.0001 | 100 | Fig. 1A |
|  |  |  |  |  |  |
| N2 unmated + OA | 14.4 ± 0.4 | -- | -- | 50 |  |
| N2 mated + OA | 15.8 ± 0.7 | 10% | 0.0457 | 100 |  |
|  |  |  |  |  |  |
| N2 unmated | 16.6 ± 0.9 | -- | -- | 67 |  |
| N2 mated | 14.9 ± 0.7 | -10% | 0.0963 | 66 |  |
|  |  |  |  |  |  |
| N2 unmated + OA | 17.8 ± 0.5 | -- | -- | 80 |  |
| N2 mated + OA | 17.0 ± 0.7 | -4% | 0.3202 | 80 |  |
|  |  |  |  |  |  |
| eat-2(ad465) unmated | 23.3 ± 1.3 | -- | -- | 75 |  |
| eat-2(ad465) mated | 22.7 ± 1.1 | -3% | 0.5056 | 72 |  |
|  |  |  |  |  |  |
| eat-2(ad465) unmated + OA | 20.8 ± 0.8 | -- | -- | 82 |  |
| eat-2(ad465) mated + OA | 23.0 ± 1.0 | 11% | 0.022 | 80 |  |
|  |  |  |  |  |  |
| N2 unmated | 16.3 ± 0.6 | -- | -- | 66 |  |
| N2 mated | 14.0 ± 0.6 | -14% | 0.0059 | 66 |  |
|  |  |  |  |  |  |
| N2 unmated + OA | 16.2 ± 0.6 | -- | -- | 66 |  |
| N2 mated + OA | 14.9 ± 0.6 | -8% | 0.1162 | 66 |  |
|  |  |  |  |  |  |
| eat-2(ad465) unmated | 22.1 ± 0.8 | -- | -- | 66 |  |
| eat-2(ad465) mated | 17.0 ± 0.8 | -23% | <0.0001 | 66 |  |
|  |  |  |  |  |  |
| eat-2(ad465) unmated + OA | 21.5 ± 0.8 | -- | -- | 66 |  |
| eat-2(ad465) mated + OA | 16.7 ± 0.8 | -22% | 0.0002 | 66 |  |
|  |  |  |  |  |  |
| N2 unmated + sDR | 23.7 ± 1.4 | -- | -- | 50 | Fig. S1D |
| N2 mated + sDR | 14.1 ± 2.0 | -41% | 0.0042 | 100 | Fig. S1D |
|  |  |  |  |  |  |
| fat-6;fat-7 unmated | 12.3 ± 0.7 | -- | -- | 60 | Fig. 1E |
| fat-6;fat-7 mated | 9.6 ± 0.4 | -22% | 0.0059 | 100 | Fig. 1E |
|  |  |  |  |  |  |
| fat-6;fat-7 unmated + OA | 15.1 ± 0.8 | -- | -- | 60 | Fig. 1F |
| fat-6;fat-7 mated + OA | 15.5 ± 0.8 | 3% | 0.7648 | 100 | Fig. 1F |
|  |  |  |  |  |  |
| fat-6;fat-7 unmated | 12.6 ± 0.7 | -- | -- | 45 |  |
| fat-6;fat-7 mated | 8.7 ± 0.3 | -30% | <0.0001 | 40 |  |
|  |  |  |  |  |  |
| fat-6;fat-7 unmated + OA | 11.8 ± 0.5 | -- | -- | 52 |  |
| fat-6;fat-7 mated + OA | 11.2 ± 0.5 | -5% | 0.4504 | 47 |  |
|  |  |  |  |  |  |
| fat-2 unmated | 18.1 ± 0.8 | -- | -- | 60 |  |
| fat-2 mated | 18.4 ± 0.6 | 2% | 0.8540 | 100 |  |
|  |  |  |  |  |  |
| fat-2 unmated + OA | 20.4 ± 0.6 | -- | -- | 60 |  |
| fat-2 mated + OA | 20.2 ± 0.7 | -1% | 0.7395 | 100 |  |
|  |  |  |  |  |  |
| fat-2 unmated | 18.6 ± 0.6 | -- | -- | 81 |  |
| fat-2 mated | 16.3 ± 0.7 | -12% | 0.0243 | 60 |  |
|  |  |  |  |  |  |
| fat-2 unmated + OA | 17.0 ± 0.5 | -- | -- | 80 |  |
| fat-2 mated + OA | 16.1 ± 0.5 | -5% | 0.2539 | 80 |  |
|  |  |  |  |  |  |
| fat-2 unmated | 17.4 ± 0.6 | -- | -- | 67 |  |
| fat-2 mated | 15.6 ± 0.6 | -11% | 0.0385 | 67 |  |
|  |  |  |  |  |  |
| fat-2 unmated + OA | 15.3 ± 0.6 | -- | -- | 66 |  |
| fat-2 mated + OA | 15.2 ± 0.7 | 0% | 0.9230 | 68 |  |
|  |  |  |  |  |  |
| fat-2 unmated | 18.1 ± 0.4 | -- | -- | 175 | Fig. 1G |
| fat-2 mated | 17.1 ± 0.4 | -5% | 0.2187 | 193 | Fig. 1G |
|  |  |  |  |  |  |
| fat-2 unmated + OA | 18.0 ± 0.4 | -- | -- | 173 | Fig. 1H |
| fat-2 mated + OA | 18.0 ± 0.4 | 0% | 0.7598 | 215 | Fig. 1H |
|  |  |  |  |  |  |
| N2 unmated | 15.4 ± 0.6 | -- | -- | 60 |  |
| fat-2(ok873) unmated | 18.2 ± 0.8 | 18% | 0.0045 | 60 |  |
|  |  |  |  |  |  |
| N2 unmated | 16.6 ± 0.9 | -- | -- | 67 |  |
| fat-2(ok873) unmated | 18.6 ± 0.6 | 12% | 0.084 | 81 |  |
|  |  |  |  |  |  |
| N2 unmated | 16.3 ± 0.6 | -- | -- | 66 |  |
| fat-2(ok873) unmated | 17.4 ± 0.6 | 7% | 0.1470 | 67 |  |
|  |  |  |  |  |  |
| N2 unmated | 15.8 ± 0.4 | -- | -- | 160 | Fig. S3A |
| fat-2(ok873) unmated | 18.1 ± 0.4 | 15% | 0.0002 | 173 | Fig. S3A |
|  |  |  |  |  |  |
| daf-2(e1370) unmated | 37.0 ± 1.9 | -- | -- | 47 | Fig. S1A |
| daf-2(e1370) mated | 19.4 ± 2.2 | -48% | <0.0001 | 60 | Fig. S1A |
|  |  |  |  |  |  |
| glp-1(e2141) unmated | 24.1 ± 1.3 | -- | -- | 48 | Fig. S1B |
| glp-1(e2141) mated | 11.6 ± 0.5 | -52% | <0.0001 | 60 | Fig. S1B |
|  |  |  |  |  |  |
|  |  |  |  |  |  |
| **Figure 2** |  |  |  |  |  |
| N2 unmated | 12.7 ± 0.6 | -- | -- | 60 | Fig. 2A |
| N2 mated | 9.4 ± 0.4 | -26% | <0.0001 | 100 | Fig. 2A |
|  |  |  |  |  |  |
| N2 unmated + OA | 14.2 ± 0.5 | -- | -- | 60 | Fig. 2B |
| N2 mated + OA | 13.3 ± 1.2 | -6% | 0.8344 | 100 | Fig. 2B |
|  |  |  |  |  |  |
| N2 unmated + LA | 14.3 ± 0.6 | -- | -- | 60 | Fig. 2C |
| N2 mated + LA | 10.2 ± 0.4 | -29% | 0.0007 | 100 | Fig. 2C |
|  |  |  |  |  |  |
| N2 unmated + VCA | 14.3 ± 0.6 | -- | -- | 60 | Fig. 2D |
| N2 mated + VCA | 10.5 ± 0.3 | -26% | <0.0001 | 100 | Fig. 2D |
|  |  |  |  |  |  |
| N2 unmated + DGLA | 15.9 ± 0.6 | -- | -- | 60 | Fig. 2E |
| N2 mated + DGLA | 9.8 ± 0.5 | -38% | <0.0001 | 100 | Fig. 2E |
|  |  |  |  |  |  |
| N2 unmated + EPA | 15.2 ± 0.5 | -- | -- | 60 | Fig. 2F |
| N2 mated + EPA | 10.3 ± 0.4 | -32% | <0.0001 | 100 | Fig. 2F |
|  |  |  |  |  |  |
| N2 unmated | 13.8 ± 0.5 | -- | -- | 69 |  |
| N2 mated | 10.4 ± 0.4 | -25% | <0.0001 | 78 |  |
|  |  |  |  |  |  |
| N2 unmated + EPA | 15.6 ± 0.6 | -- | -- | 65 |  |
| N2 mated + EPA | 11.1 ± 0.7 | -29% | <0.0001 | 75 |  |
|  |  |  |  |  |  |
| N2 unmated + LA | 15.0 ± 0.5 | -- | -- | 67 |  |
| N2 mated + LA | 10.7 ± 0.5 | -29% | <0.0001 | 77 |  |
|  |  |  |  |  |  |
| N2 unmated + VCA | 15.0 ± 0.6 | -- | -- | 67 |  |
| N2 mated + VCA | 11.5 ± 0.8 | -23% | 0.0001 | 75 |  |
|  |  |  |  |  |  |
| N2 unmated + DGLA | 14.9 ± 0.5 | -- | -- | 67 |  |
| N2 mated + DGLA | 10.5 ± 0.7 | -30% | <0.0001 | 74 |  |
|  |  |  |  |  |  |
|  |  |  |  |  |  |
| **Figure 4** |  |  |  |  |  |
| N2 unmated | 16.7 ± 0.8 | -- | -- | 60 | Fig. 4A |
| N2 2hr mated | 17.8 ± 0.5 | 7% | 0.5900 | 100 | Fig. 4A |
|  |  |  |  |  |  |
| N2 unmated + OA | 17.4 ± 0.7 | -- | -- | 60 | Fig. 4C |
| N2 2hr mated + OA | 17.7 ± 0.6 | 2% | 0.7676 | 100 | Fig. 4C |
|  |  |  |  |  |  |
| fog-2(q71) unmated | 18.4 ± 1.2 | -- | -- | 60 | Fig. 4B |
| fog-2(q71) 2hr mated | 14.0 ± 1.0 | -24% | 0.0044 | 100 | Fig. 4B |
|  |  |  |  |  |  |
| fog-2(q71) unmated + OA | 16.4 ± 0.8 | -- | -- | 60 | Fig. 4D |
| fog-2(q71) 2hr mated + OA | 16.2 ± 0.9 | -1% | 0.5910 | 100 | Fig. 4D |
|  |  |  |  |  |  |
| hlh-30(tm1978) unmated | 16.6 ± 0.8 | -- | -- | 60 | Fig. 4E |
| hlh-30(tm1978) 2hr mated | 14.3 ± 0.4 | -14% | 0.0117 | 100 | Fig. 4E |
|  |  |  |  |  |  |
| hlh-30(tm1978) unmated + OA | 19.4 ± 0.5 | -- | -- | 60 | Fig. 4F |
| hlh-30(tm1978) 2hr mated + OA | 18.8 ± 0.4 | -3% | 0.4357 | 100 | Fig. 4F |
|  |  |  |  |  |  |
| N2 unmated | 13.2 ± 0.4 | -- | -- | 58 |  |
| N2 2hr mated | 12.8 ± 0.4 | -3% | 0.6223 | 61 |  |
|  |  |  |  |  |  |
| N2 unmated + OA | 14.1 ± 0.5 | -- | -- | 53 |  |
| N2 2hr mated + OA | 12.5 ± 0.4 | -11% | 0.0675 | 58 |  |
|  |  |  |  |  |  |
| fog-2(q71) unmated | 18.0 ± 0.5 | -- | -- | 115 |  |
| fog-2(q71) 2hr mated | 15.3 ± 0.5 | -15% | <0.0001 | 58 |  |
|  |  |  |  |  |  |
| fog-2(q71) unmated + OA | 16.5 ± 0.7 | -- | -- | 48 |  |
| fog-2(q71) 2hr mated + OA | 15.8 ± 0.6 | -4% | 0.5099 | 61 |  |
|  |  |  |  |  |  |
| hlh-30(tm1978) unmated | 12.7 ± 0.3 | -- | -- | 63 |  |
| hlh-30(tm1978) 2hr mated | 10.2 ± 0.4 | -20% | <0.0001 | 65 |  |
|  |  |  |  |  |  |
| hlh-30(tm1978) unmated + OA | 13.6 ± 0.3 | -- | -- | 63 |  |
| hlh-30(tm1978) 2hr mated + OA | 12.9 ± 0.5 | -5% | 0.7194 | 66 |  |
|  |  |  |  |  |  |
| *C. remanei* unmated | 22.6 ± 1.2 | -- | -- | 60 | Fig. 4G |
| *C. remanei* mated | 18.2 ± 0.8 | -20% | 0.0029 | 60 | Fig. 4G |
|  |  |  |  |  |  |
| *C. remanei* unmated + OA | 21.9 ± 1.4 | -- | -- | 60 | Fig. 4G |
| *C. remanei* mated + OA | 25.3 ± 0.9 | 15% | 0.3266 | 60 | Fig. 4G |
|  |  |  |  |  |  |
| *C. remanei* unmated + OA | 29.8 ± 1.5 | -- | -- | 60 |  |
| *C. remanei* mated + OA | 28.7 ± 1.2 | -4% | 0.3331 | 60 |  |
|  |  |  |  |  |  |
| N2 unmated | 16.6 ± 0.5 | -- | -- | 49 | Fig. 4H |
| N2 mated | 11.8 ± 0.5 | -29% | <0.0001 | 80 | Fig. 4H |
|  |  |  |  |  |  |
| N2 unmated + OA | 17.9 ± 0.6 | -- | -- | 50 | Fig. 4H |
| N2 mated + OA | 18.1 ± 0.6 | 1% | 0.9466 | 80 | Fig. 4H |
|  |  |  |  |  |  |
| N2 unmated | 14.2 ± 0.5 | -- | -- | 50 | Fig. S3B |
| N2 mated | 9.5 ± 0.3 | -33% | <0.0001 | 100 | Fig. S3B |
| N2 mated + 0.8 mM OA | 12.2 ± 0.6 | -14% | 0.1435 | 100 | Fig. S3B |
| N2 mated + 2.0 mM OA | 13.8 ± 0.7 | -3% | 0.8558 | 100 | Fig. S3B |
|  |  |  |  |  |  |
